# Supplementary material for: Fine-scale patterns of SARS-CoV-2 spread from identical pathogen sequences
Source: Nature. 2025 Mar 5;640(8057):176–85. doi: 10.1038/s41586-025-08637-4 (PMC11964829; doi:10.1038/s41586-025-08637-4)
Supplement: Supplementary file 1 — Supplementary Figs. 1–25, Supplementary Tables 1–3, Supplementary Note 1 and Supplementary References. [file 41586_2025_8637_MOESM1_ESM.pdf]

nature portfolio

https://doi.org/10.1038/s41586-025-08637-4

Supplementary information

Fine-scale patterns of SARS-CoV-2 spread from identical pathogen sequences

In the format provided by the authors and unedited

Supplementary information: Fine-scale patterns of SARS-CoV-2 spread from identical pathogen sequences

Cécile Tran-Kiem[1,*], Miguel I. Paredes[1,2], Amanda C. Perofsky[3,4], Lauren A. Frisbie[5], Hong Xie[6], Kevin Kong[6], Amelia Weixler[6], Alexander L. Greninger[1,6], Pavitra Roychoudhury[1,6], JohnAric M. Peterson[5], Andrew Delgado[5], Holly Halstead[5], Drew MacKellar[5], Philip Dykema[5], Luis Gamboa[3], Chris D. Frazar[7], Erica Ryke[7], Jeremy Stone[3], David Reinhart[3], Lea Starita[3,7], Allison Thibodeau[5], Cory Yun[5], Frank Aragona[5], Allison Black[5], Cécile Viboud[4] & Trevor Bedford[1,8]

[1]Vaccine and Infectious Diseases Division, Fred Hutchinson Cancer Center, Seattle, WA, USA, [2]Department of Epidemiology, University of Washington, Seattle, WA, USA, [3]Brotman Baty Institute, University of Washington, Seattle, WA, USA, [4]Fogarty International Center, National Institutes of Health, Bethesda, MD, USA, [5]Washington State Department of Health, Shoreline, WA, USA, [6]Department of Laboratory Medicine and Pathology, University of Washington, Seattle, WA, USA, [7]Department of Genome Sciences, University of Washington, Seattle, WA, USA, [8]Howard Hughes Medical Institute, Seattle, WA, USA, *To whom correspondence should be addressed; E-mail: ctrankie@fredhutch.org

List of supplementary figures

S1 Permutation test to explore the spatio-temporal signal in clusters of identical SARS-CoV-2 sequences. 3
S2 Relative risk of observing identical sequences in two counties. 3
S3 Relative risk of observing pairs of identical sequences between counties. 5
S4 Impact of subsampling on the significance of the association between the relative risk of identical sequences and whether counties and ZCTAs are adjacent or not. 6
S5 Distribution of the delay between sequence collection within pairs of identical sequences collected between Eastern and Western WA and across epidemic waves. 7
S6 Comparison between the number of directed commuting flows and the number of directed visits between two counties. 7
S7 Expected relationship between the RR of observing identical sequences in two age groups and the RR of contacts between these age groups. 8
S8 Geographical regions used to aggregate counties. 9
S9 Comparison between the relative risk of observing identical sequences and the relative risk of movement at the region level. 10
S10 Relationship between the relative risk of observing identical sequences in two regions and the relative risk of movement between these regions obtained from mobile phone mobility data across epidemic waves 11
S11 Comparison of the total number of visits between pairs of WA counties across the 4 epidemic waves during our study period. 12
S12 Comparison of connectivity metrics across the Eastern / Western WA border among counties located on the border. 13

S13 Patterns of occurrence of pairs of identical sequences between ZCTAs in Pierce and Mason counties, the two counties that are home of WA female prisons. . . . 14
S14 Median delay between the dates of sequence collection within pairs of identical sequences 15
S15 Sensitivity analysis on the timing of pairs identical sequences between age groups using symptom onset dates 16
S16 Ratio between the relative risk of observing identical sequences within a given vaccination group (denoted V_1) and between two vaccination groups (denoted V_1 and V_2). 16
S17 Sensitivity analysis for our transmission direction analysis relying on clusters of identical sequences observed only in two groups. 17
S18 Impact of dataset size on the number of clusters of identical sequences and the number of sequences with another identical sequence in the dataset. 18
S19 Impact of the number of groups included in the analysis on the dataset size required for the error in the relative risk of observing identical sequences to be lower than 10%. 19
S20 Comparisons between county census population sizes and SafeGraph panel sizes in Washington state, 2020 – 2022. 20
S21 County-level bias of SafeGraph data in Washington state does not correlate with A. census population size, B. SafeGraph panel sizes in individual counties relative to WA state ("county observed proportion"), or C. census urban-rural classification, 2020 – 2022. 21
S22 Empirical distribution of the delay between symptom onset and sequence collection by age (rows), period (columns) and geographic region (colours). 22
S23 Characteristics of clusters of identical sequences across the study period. 23
S24 Time-series of COVID-19 cases in WA over the study periods. 23
S25 Illustration of the downsampling strategy used to quantify the amount of data required to compute relative risks. 24

List of supplementary tables

S1 Performance of Discrete Trait Analysis (DTA) and our relative risk metric (RR) in quantifying migration patterns. 25
S2 Comparison of the relative of risk of observing identical sequences at the ZCTA level by adjacency level. 25
S3 Characteristics of WA male prisons. 26

List of supplementary texts

S1 Relationship between the number of transmission pairs and the number of pairs with timing consistent with a transmission direction 27

References **28**

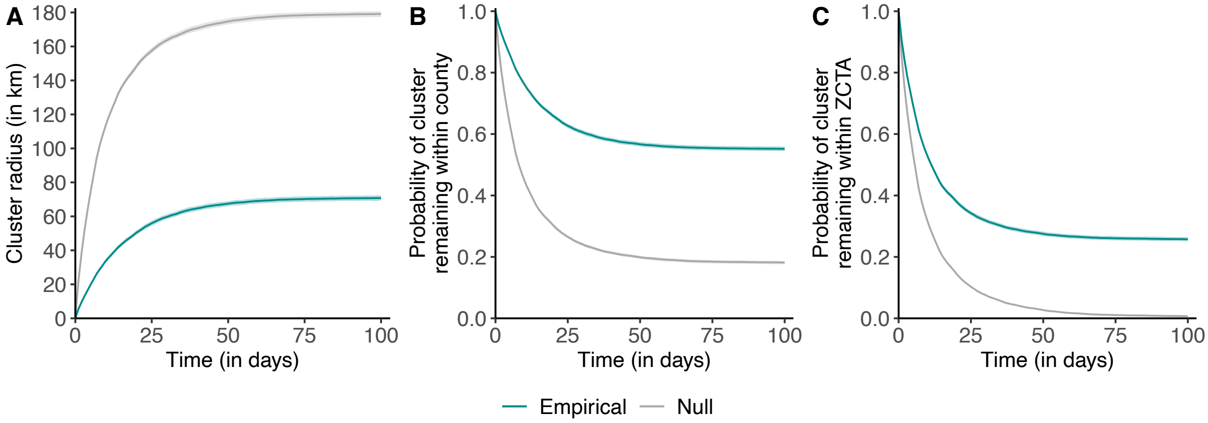

Figure S1. Permutation test to explore the spatio-temporal signal in clusters of identical SARS-CoV-2 sequences. **A.** Radius of clusters of identical sequences as a function of time since first sequence collection. **B.** Probability for all sequences within a cluster of identical sequences of remaining in the same county as a function of time since first sequence collection. **C.** Probability for all sequences within a cluster of identical sequences of remaining in the same ZCTA as a function of time since first sequence collection. The grey shaded areas correspond to 95% confidence intervals of a null distribution generated from 100 simulations where the geographical location of sequences from WA Sentinel surveillance are permuted. The grey lines correspond to the medians of the simulated null distributions.

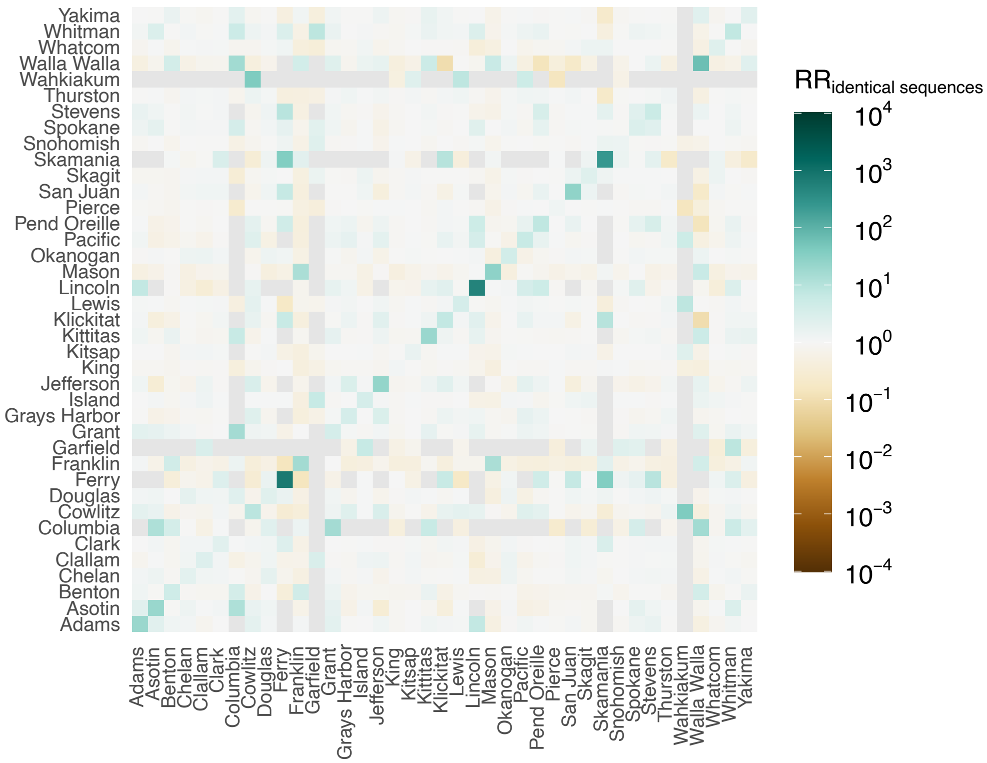

Figure S2. Relative risk of observing identical sequences in two counties. Grey squares correspond to pairs of counties between which no pair of identical sequences is observed during the study period.

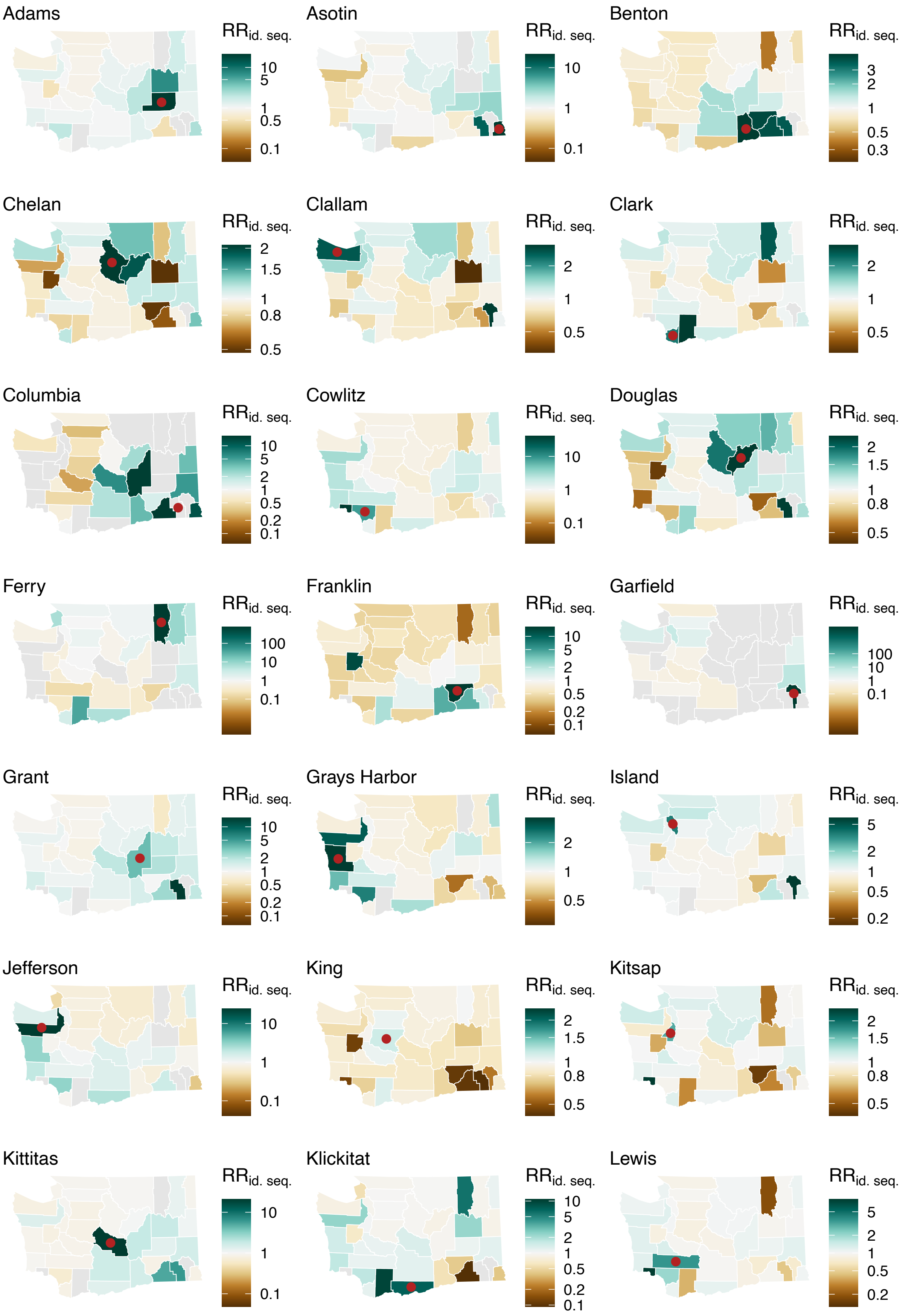
Adams
RRid. seq.
10
5
1
0.5
0.1
Asotin
RRid. seq.
10
1
0.1
Benton
RRid. seq.
3
2
1
0.5
0.3
Chelan
RRid. seq.
2
1.5
1
0.8
0.5
Clallam
RRid. seq.
2
1
0.5
Clark
RRid. seq.
2
1
0.5
Columbia
RRid. seq.
10
5
2
1
0.5
0.2
0.1
Cowlitz
RRid. seq.
10
1
0.1
Douglas
RRid. seq.
2
1.5
1
0.8
0.5
Ferry
RRid. seq.
100
10
1
0.1
Franklin
RRid. seq.
10
5
2
1
0.5
0.2
0.1
Garfield
RRid. seq.
100
10
1
0.1
Grant
RRid. seq.
10
5
2
1
0.5
0.2
0.1
Grays Harbor
RRid. seq.
2
1
0.5
Island
RRid. seq.
5
2
1
0.5
0.2
Jefferson
RRid. seq.
10
1
0.1
King
RRid. seq.
2
1.5
1
0.8
0.5
Kitsap
RRid. seq.
2
1.5
1
0.8
0.5
Kittitas
RRid. seq.
10
1
0.1
Klickitat
RRid. seq.
10
5
2
1
0.5
0.2
0.1
Lewis
RRid. seq.
5
2
1
0.5
0.2

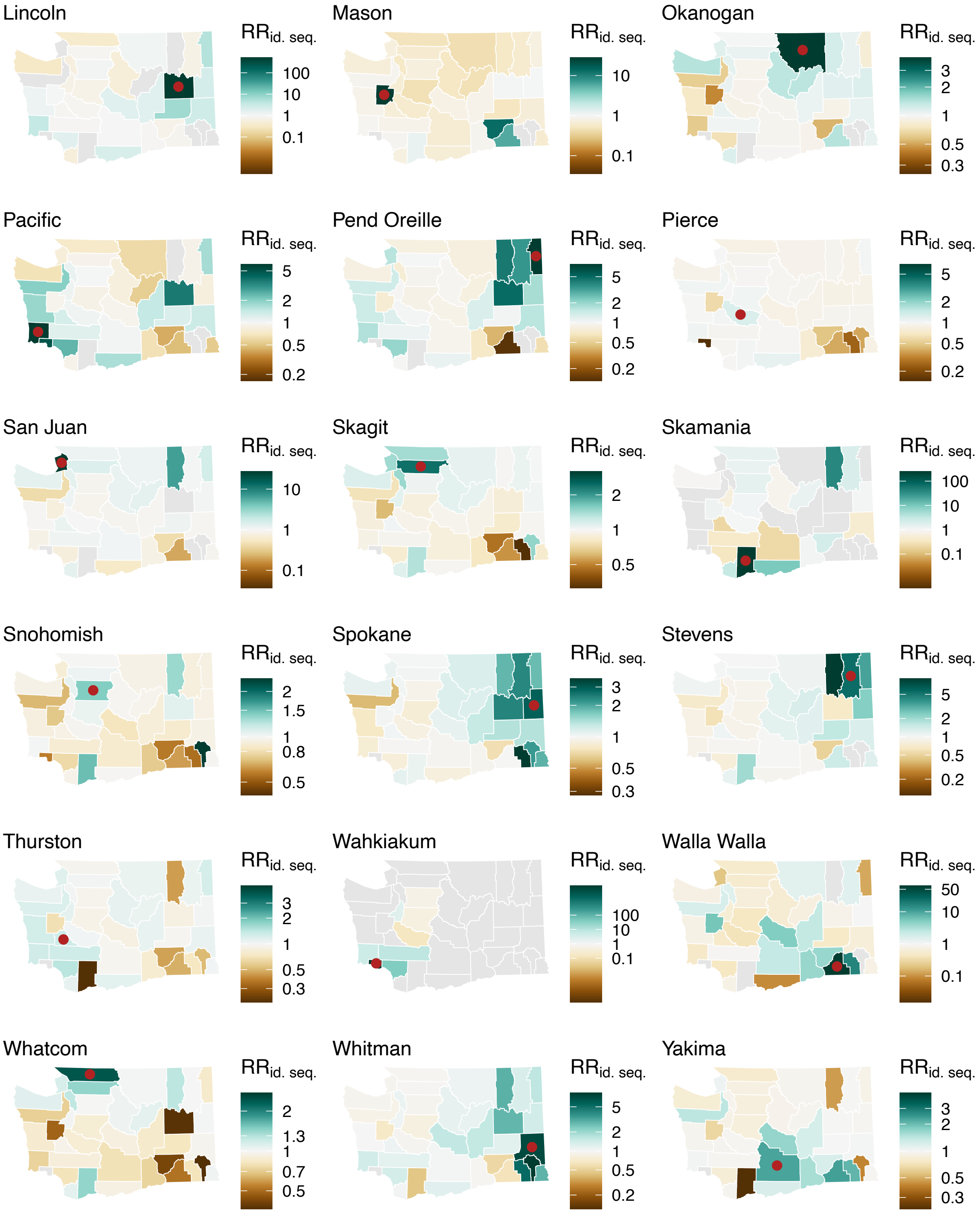

Figure S3. Relative risk of observing pairs of identical sequences between counties. On each map, we represent the relative risk of observing pairs of identical sequences in the county indicated by a red point (map title) and all the other counties in Washington state. Areas are coloured in grey when no pair of identical sequences is observed. To increase readability, each map has its own colour scale. Maps are generated using shapefiles from the US Census Bureau [1].

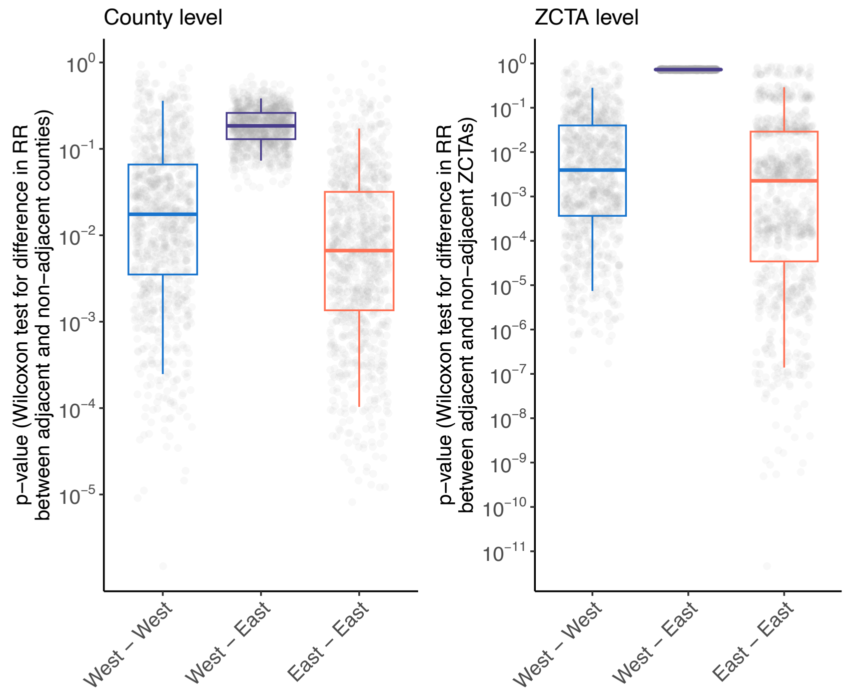

Figure S4. Impact of subsampling on the significance of the association between the relative risk of identical sequences and whether counties and ZCTAs are adjacent or not. We investigate whether our conclusions regarding the significance of the association between the relative risk of identical sequences falling in two distinct counties / ZCTAs and their adjacency status (adjacent / non-adjacent) can be impacted by the number of pairs of counties involved in the comparison (within Eastern WA, within Western WA and between Eastern and Western WA). At the county level, we subsample the pairs of counties involved in these 3 comparisons to 12 adjacent pairs of counties (number of pairs of adjacent counties between Eastern and Western WA) and 132 non-adjacent pairs of counties (number of pairs of non-adjacent counties within Western WA). This ensures that all comparisons are performed on the same number of pairs of counties. On each subsampled dataset, we compute the p-value from a Wilcoxon test evaluating differences between the relative risk of observing identical sequences in adjacent and non-adjacent counties. This is done for 1,000 subsampled datasets. Boxplots indicate p-values obtained across these different subsampling iterations (5%, 25%, 50%, 75% and 95% quantiles). We perform a similar analysis at the ZCTA level.

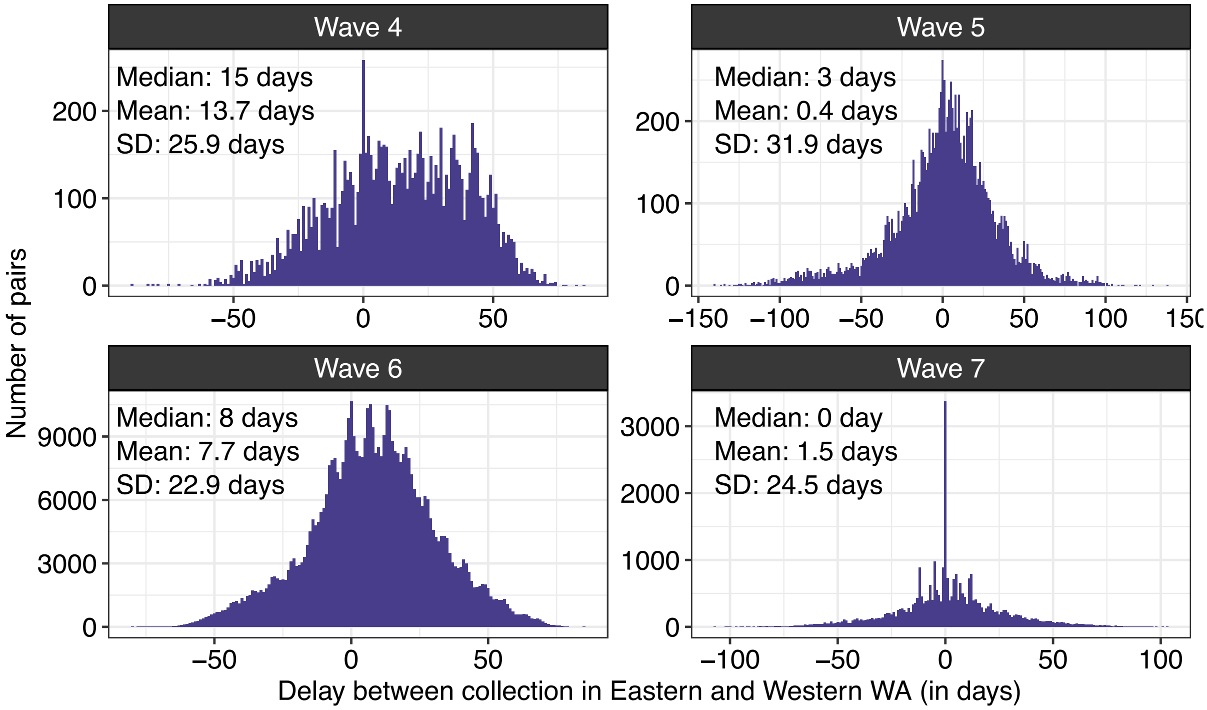

Figure S5. Distribution of the delay between sequence collection within pairs of identical sequences collected between Eastern and Western WA and across epidemic waves.

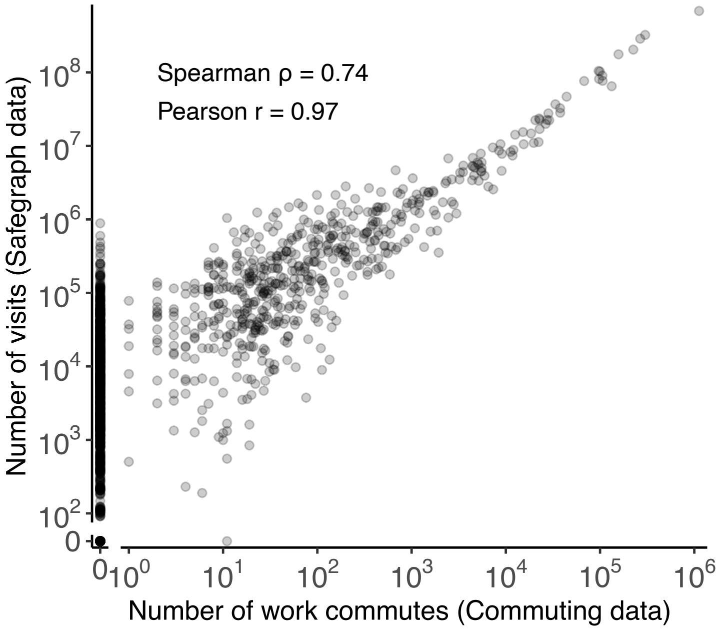

Figure S6. Comparison between the number of directed commuting flows and the number of directed visits between two counties. The number of work commutes is extracted from [2]. The number of visits is estimated using Safegraph *Weekly patterns* mobility data. The comparison is done by matching the origin county in the mobile phone data to the residence county in the workflow data and the destination county in the mobile phone data to the workplace county in the workflow data.

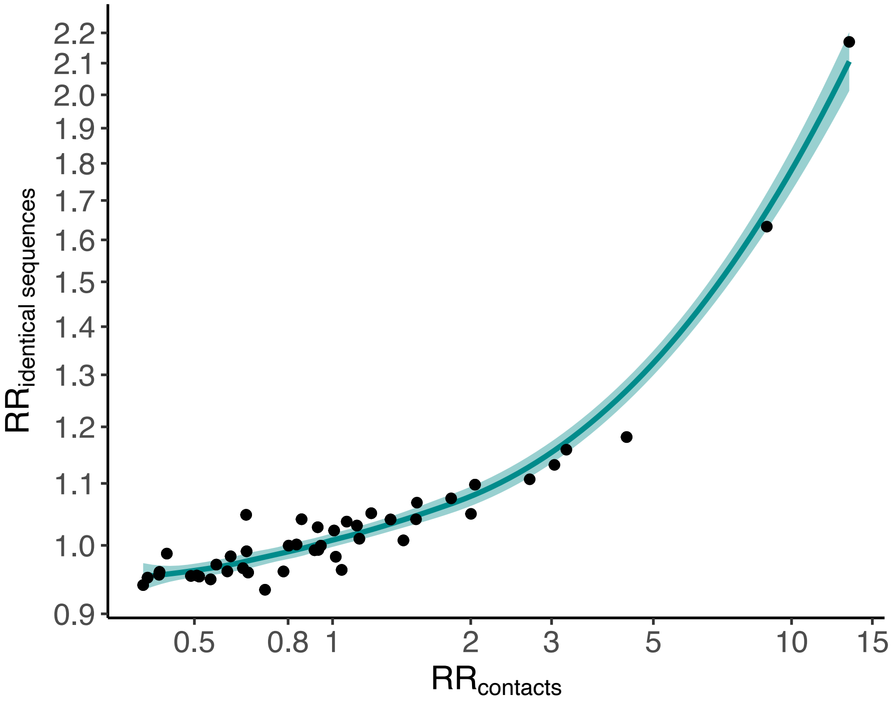

Figure S7. Expected relationship between the RR of observing identical sequences in two age groups and the RR of contacts between these age groups. These results were obtained by simulating 10^5 clusters of identical sequences from a branching process with mutations [3] using a Poisson offspring distribution. The simulation was parametrised by a reproduction number of 1.2, a probability that an infector and an infectee have the same consensus sequence of 0.7 and a sequencing fraction of 10%.

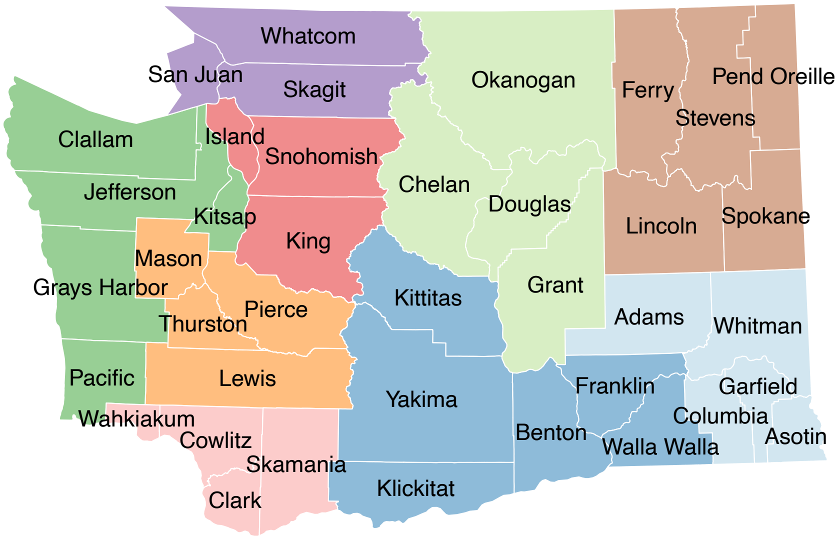

Figure S8. Geographical regions used to aggregate counties. Dark green: Peninsula/Coastal. Orange: South Puget Sound. Purple: Northwest. Red: North Puget Sound. Pink: Southwest. Dark blue: South Central. Light blue: Southeast.Brown: Northeast. Light green: North Central. Maps were generated using shapefiles from the US Census Bureau [1].

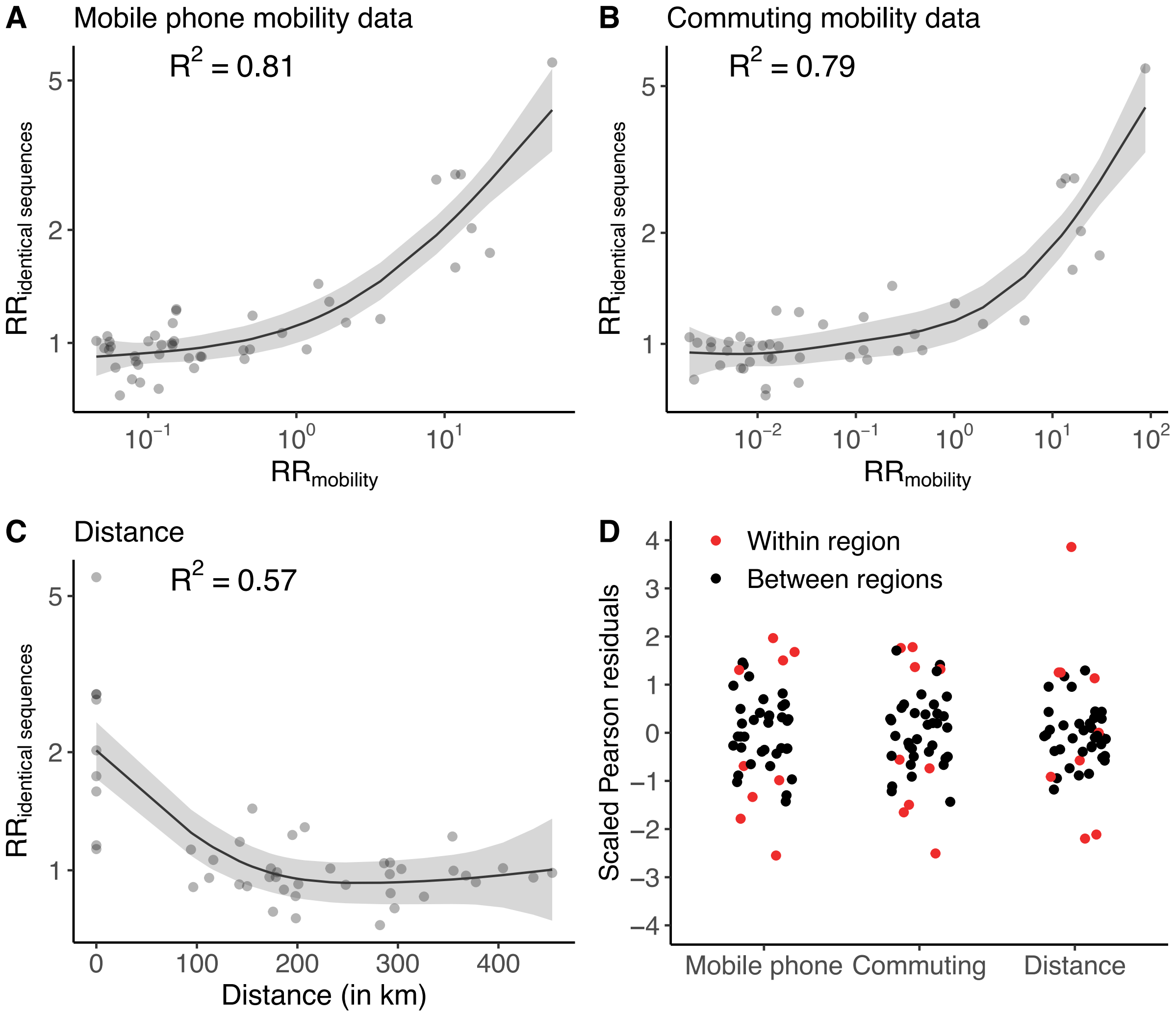

Figure S9. Comparison between the relative risk of observing identical sequences and the relative risk of movement at the region level. **A.** Relationship between the relative risk of observing identical sequences in two regions and the relative risk of movement between these regions as obtained from mobile phone mobility data. **B.** Relationship between the relative risk of observing identical sequences in two regions and the relative risk of movement between these regions as obtained from workflow mobility data. **C.** Relationship between the relative risk of observing identical sequences in two regions and the euclidean distance between region centroids. **D.** Scaled Pearson residuals of the GAM between the relative risk of observing identical sequences in two regions and (i) the relative risk of movement from commuting data, (ii) the relative risk of movement from mobile phone data and (iii) the geographic distance between regions' centroids. The trend lines correspond to predicted relative risk of observing identical sequences in two regions from each GAM. R^2 indicates the variance explained by each GAM.

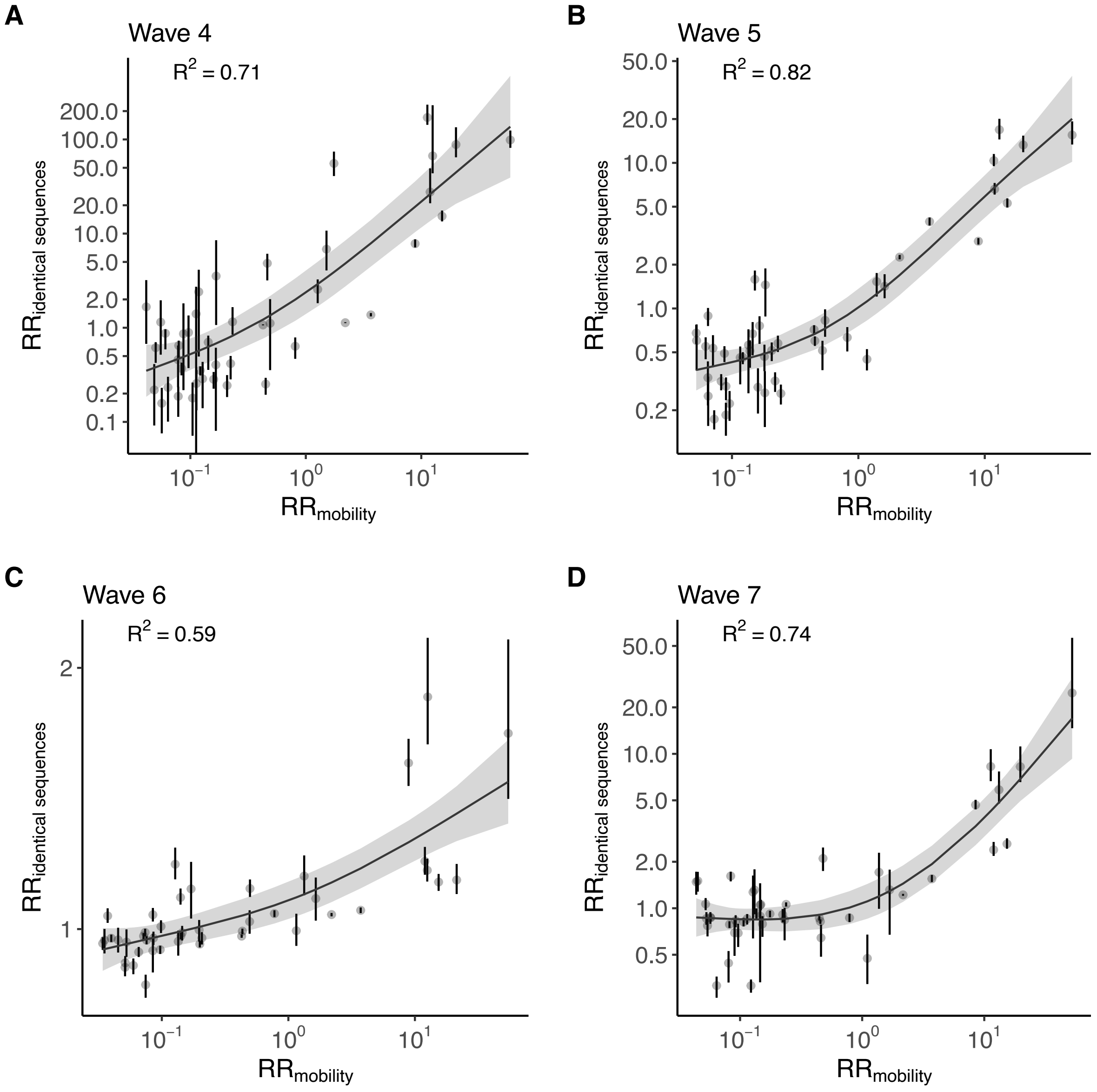

Figure S10. Relationship between the relative risk of observing identical sequences in two regions and the relative risk of movement between these regions obtained from mobile phone mobility data across epidemic waves **A.** Wave 4. **B.** Wave 5. **C.** Wave 6. **D.** Wave 7. Vertical segments indicate 95% subsampling confidence intervals. The trend lines correspond to predicted relative risk of observing identical sequences in two regions from a GAM. R^2 indicates the variance explained by each GAM.

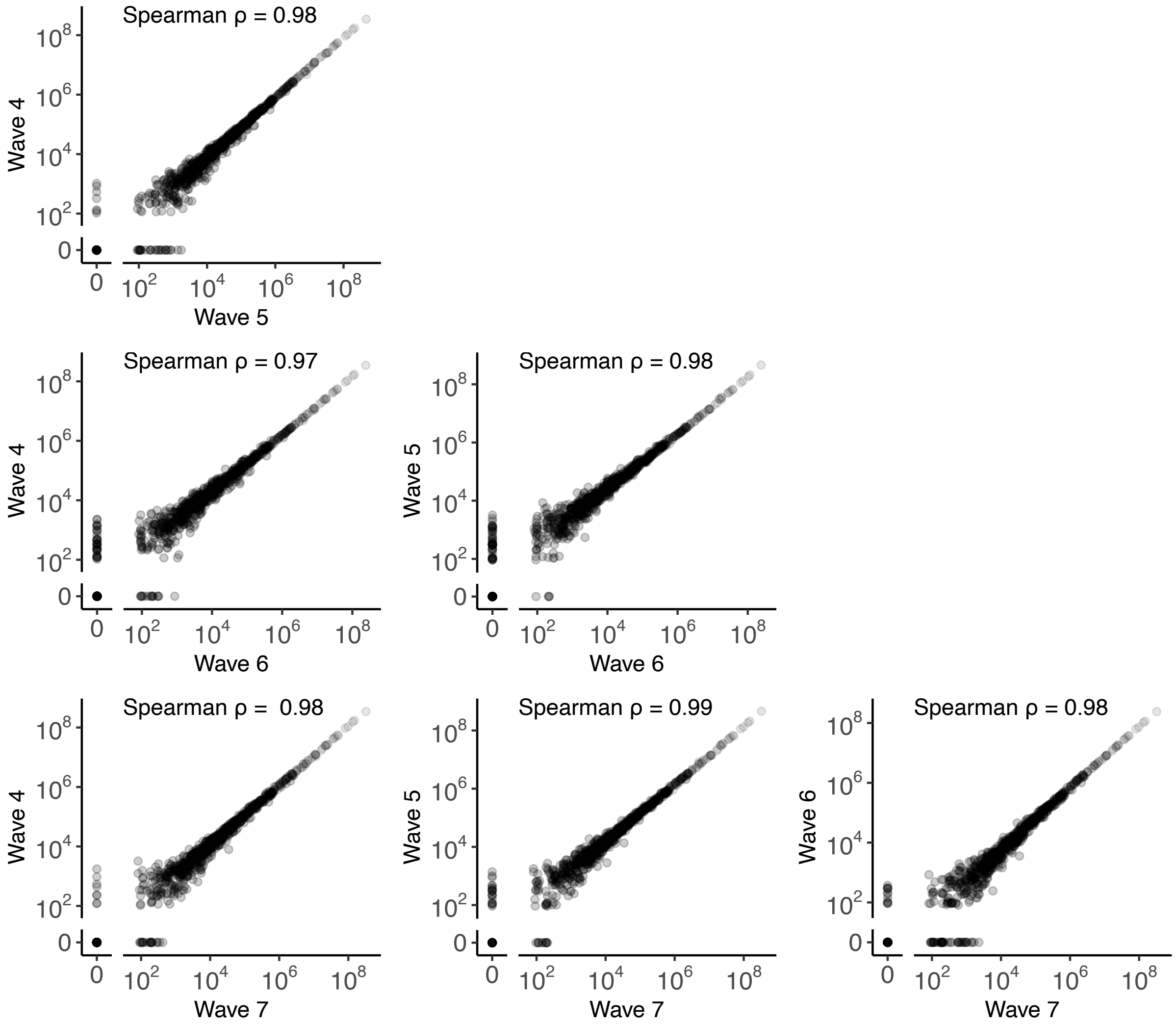

Figure S11. Comparison of the total number of visits between pairs of WA counties across the 4 epidemic waves during our study period. Points indicate the total number of visits between pairs of counties over the study periods labeled on the plot axes.

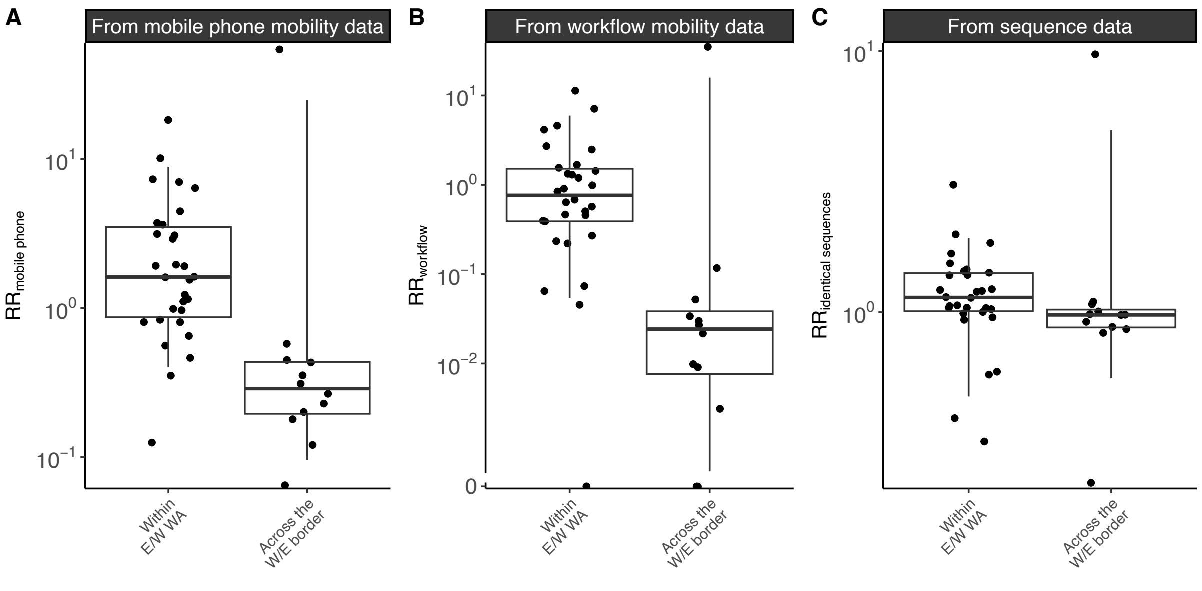

Figure S12. Comparison of connectivity metrics across the Eastern / Western WA border among counties located on the border. **A.** Relative risk of movement from mobile phone data across the border or within Eastern / Western WA (p-value for Wilcoxon rank sum test of $6.1 \cdot 10^{-5}$). **B.** Relative risk of movement from commuting data across the border or within Eastern / Western WA (p-value for Wilcoxon rank sum test of $1.6 \cdot 10^{-4}$). **C.** Relative risk of observing identical sequences across the border or within Eastern / Western WA (p-value for Wilcoxon rank sum test of $2.5 \cdot 10^{-2}$). In this analysis, we only consider WA counties along the W/E border.

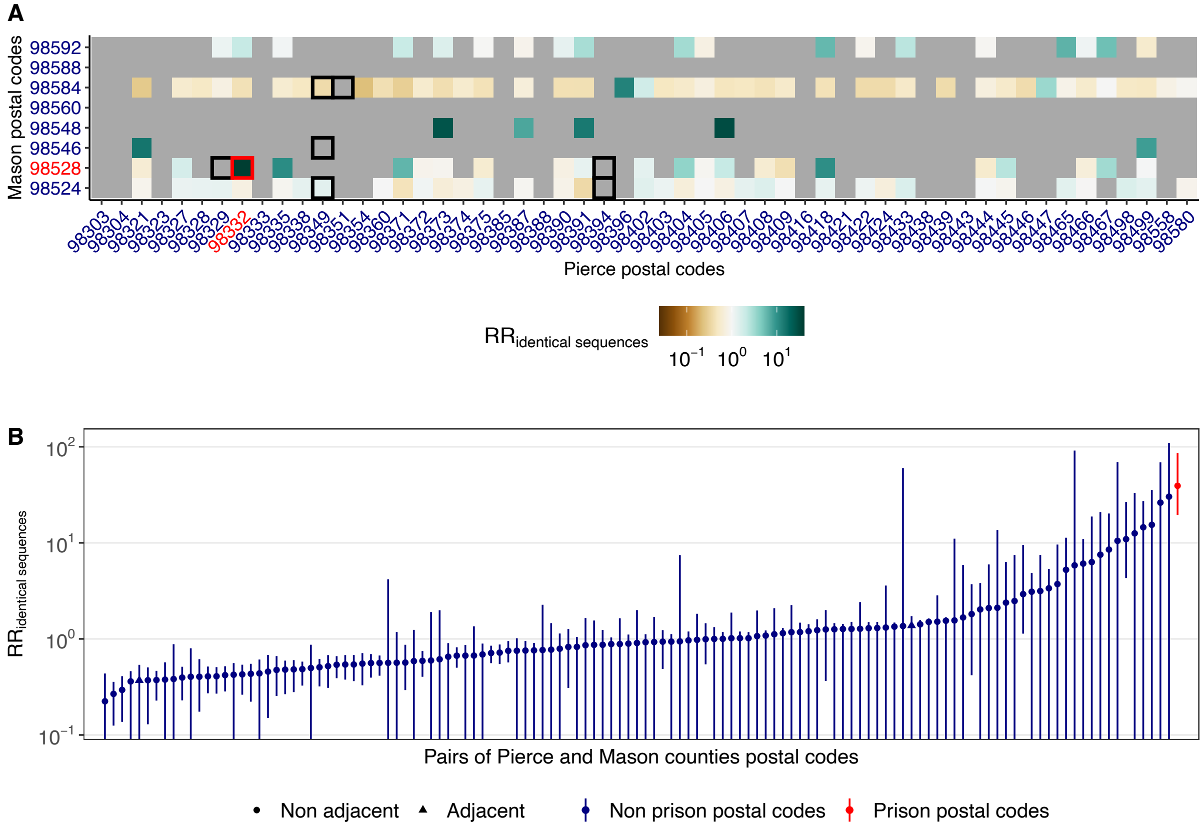

Figure S13. Patterns of occurrence of pairs of identical sequences between ZCTAs in Pierce and Mason counties, the two counties that are home of WA female prisons. A. Relative risk of observing identical sequences between ZCTAs in Mason and Pierce counties. Black squares indicate adjacent ZCTAs. ZCTAs in red correspond to postal codes that are the home of female prisons. **B.** Relative risk of observing identical sequences between Mason and Pierce counties ZCTAs ordered by increasing values. Vertical segments correspond to 95% subsampling confidence intervals.

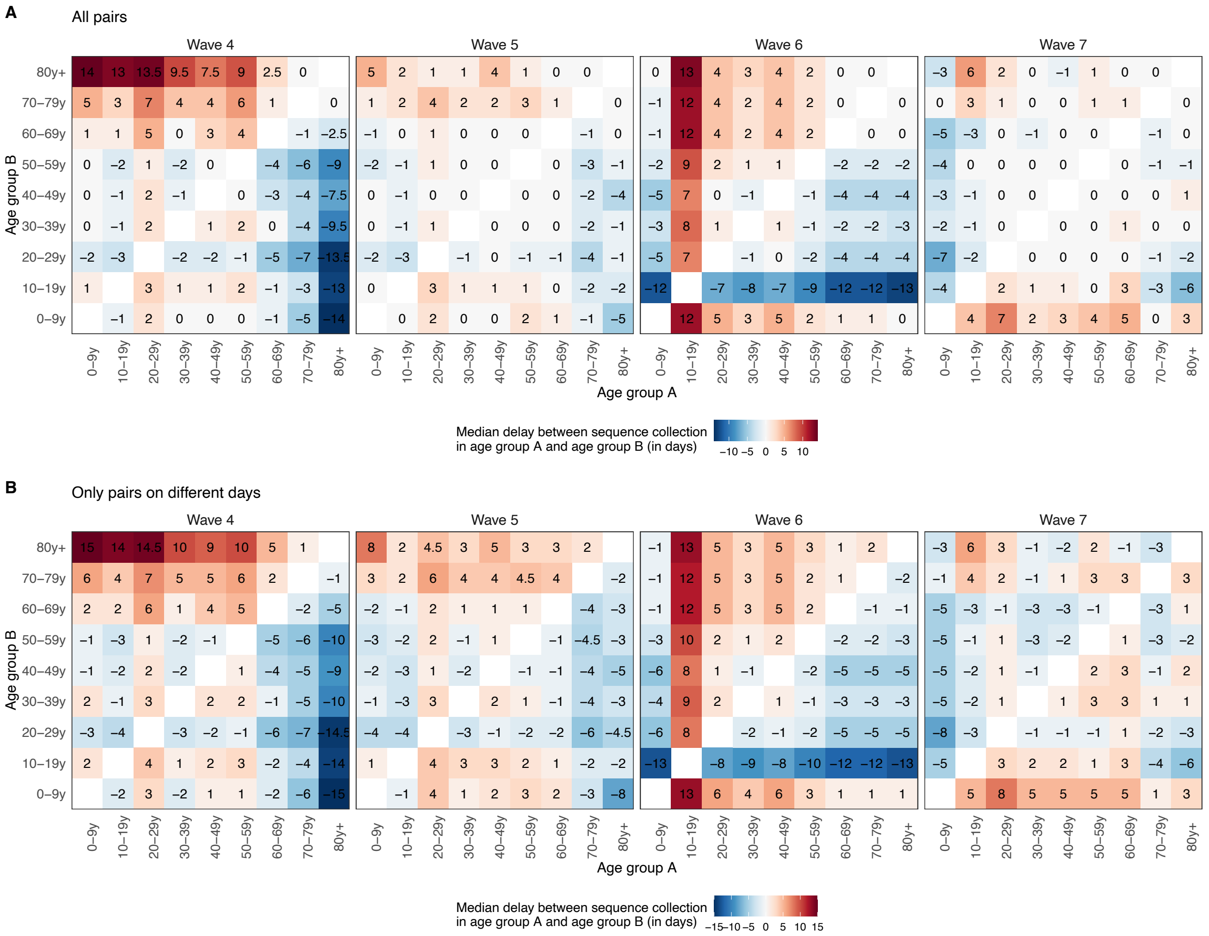

Figure S14. Median delay between the dates of sequence collection within pairs of identical sequences A. considering all pairs of identical sequences collected in two age groups and **B.** considering only pairs of identical sequences collected on different days in two age groups.

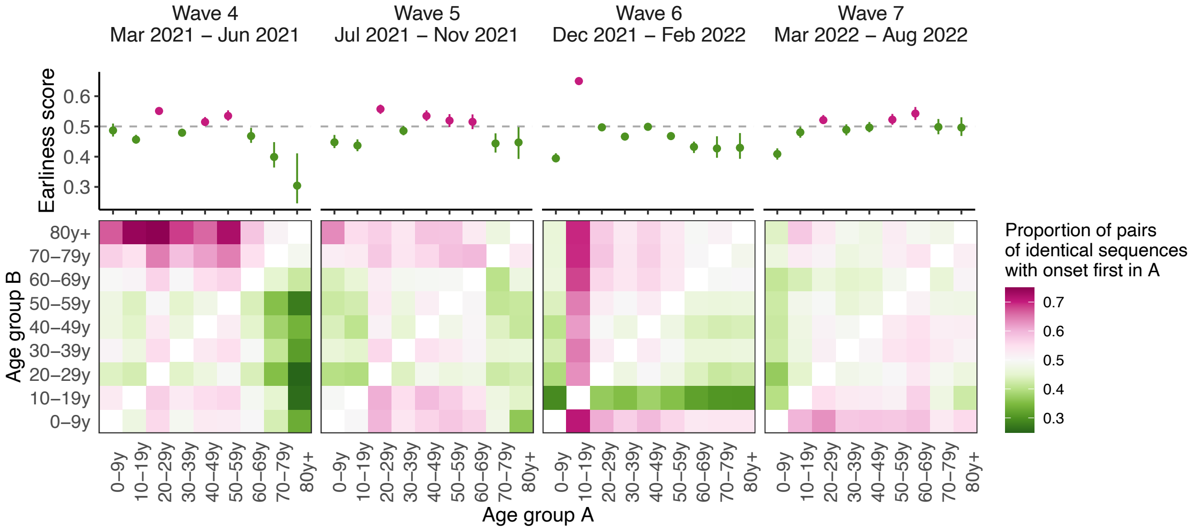

Figure S15. Sensitivity analysis on the timing of pairs identical sequences between age groups using symptom onset dates Median proportion of pairs of identical sequences with onset dates in age groups A before age group B across different epidemic waves from 1,000 imputed datasets (heatmaps). The dots plots depict the median earliness scores of age group A across 1,000 imputed datasets for the different epidemic waves. Vertical segments indicate uncertainty range around earliness scores (see Methods).

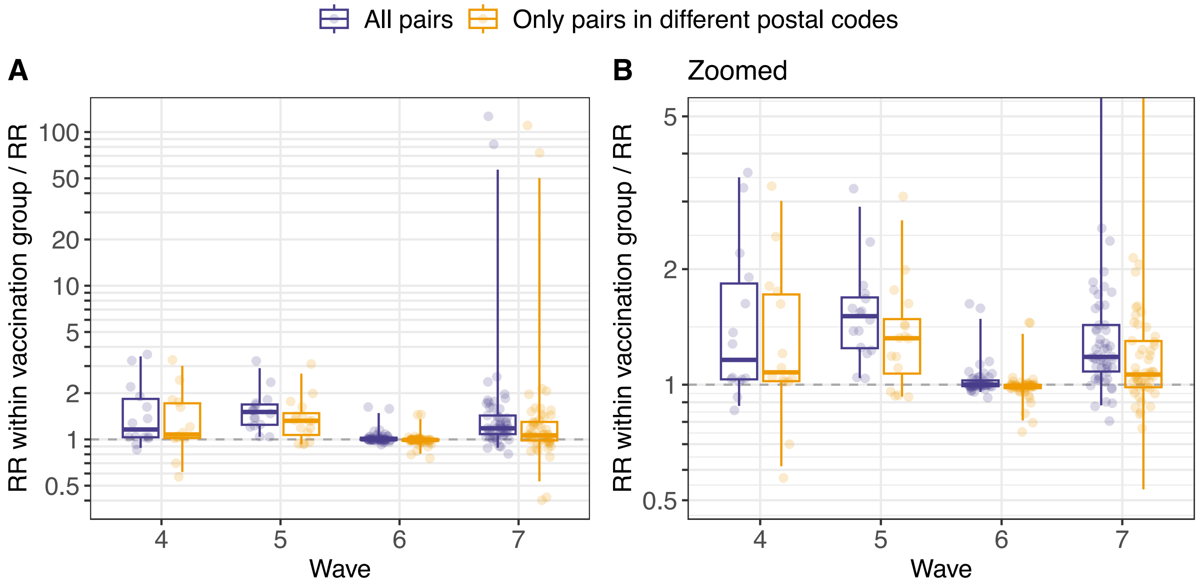

Figure S16. Ratio between the relative risk of observing identical sequences within a given vaccination group (denoted V_1) and between two vaccination groups (denoted V_1 and V_2). Values above 1 indicate that pairs of identical sequences tend to be enriched in pairs observed within the same vaccination group. The analysis is restricted to pairs observed within the same age group. Each point correspond to the ratio computed for a given pair of vaccination status (V_1, V_2) and age group. Boxplots indicate the 2.5%, 25%, 50%, 75% and 97.5% percentiles.

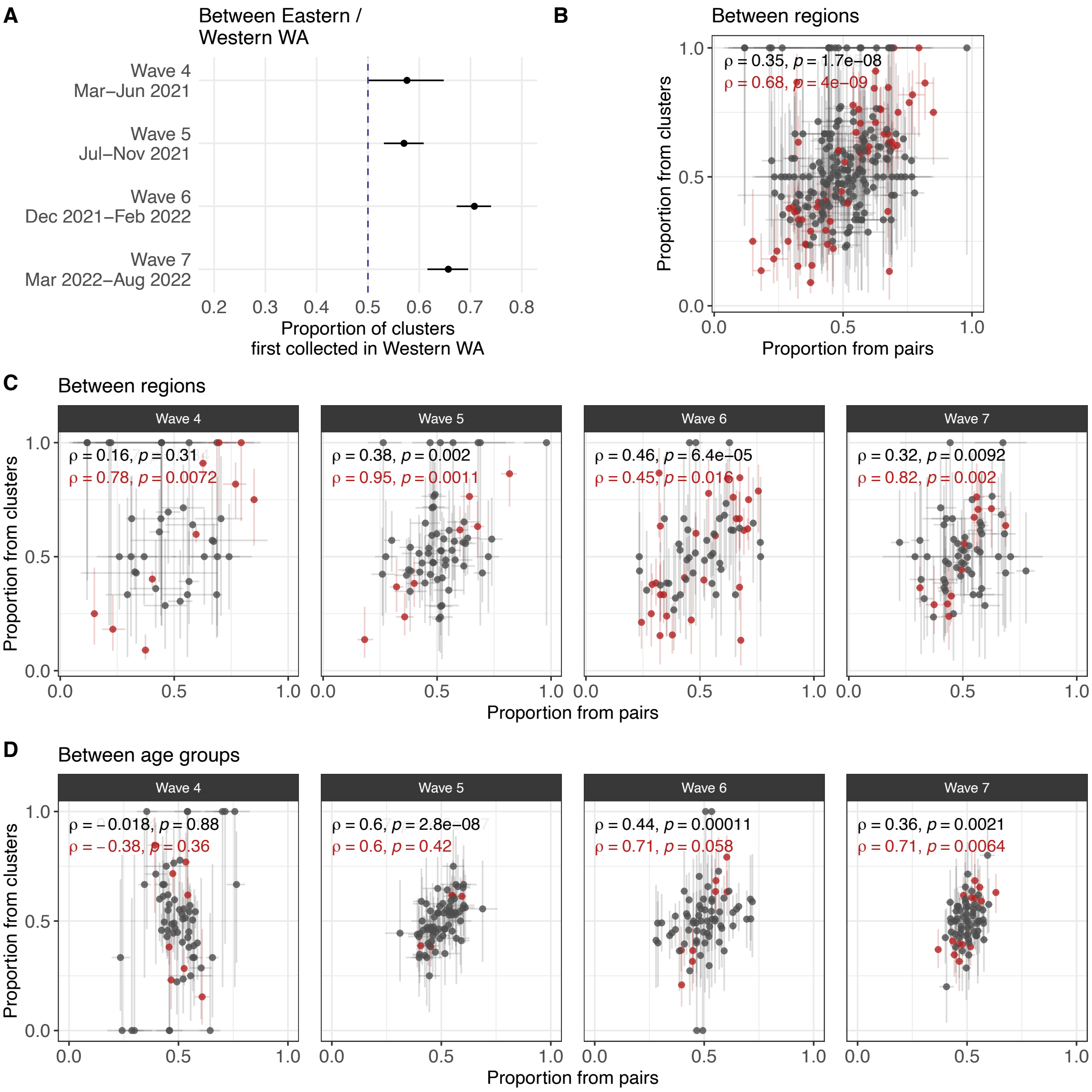

Figure S17. Sensitivity analysis for our transmission direction analysis relying on clusters of identical sequences observed only in two groups. **A.** Proportion of clusters first collected in Western WA among clusters observed in Eastern and Western WA and across pandemic waves. Like in Figure 2G, these proportions are all greater than 0.5. **B.** Sensitivity analysis at the regional level (Figure S8) comparing the proportion from pairs and the proportion from clusters. **C.** Sensitivity analysis at the regional level comparing the proportion from pairs and the proportion from clusters across waves. **D.** Sensitivity analysis at the age level comparing the proportion from pairs and the proportion from clusters across waves. For wave 4, the cluster based analysis relies on less than 10 clusters in 13 out of 36 pairs of age groups, which could explain the poor correlation. Segments indicate 95% CIs around proportions. In B, C and D, the colour red depicts points for which the CIs don't cross 0.5 for both the proportion from clusters and the proportion from clusters. We report in red the Spearman correlation coefficient with p-values for these red points and in black for all the points.

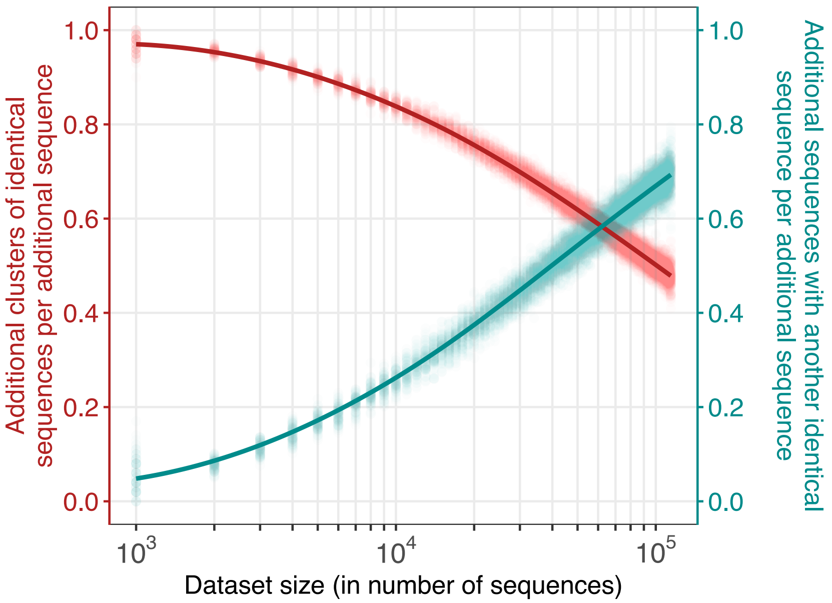

Figure S18. Impact of dataset size on the number of clusters of identical sequences and the number of sequences with another identical sequence in the dataset. We generate this figure by considering sequence datasets of increasing sizes, ranging between 10^2 and the 114,298 (the size of our WA dataset) with an increment of 10^2 between 10^2 and 10^3 and an increment of 10^3 above 10^3. We run 100 simulations where we first downsample 10^2 sequences from our full dataset and then incrementally include more sequences (drawn from the total remaining sequences not yet included). At every step, we compute the additional number of clusters of identical sequences per additional sequences (red) as well as the additional number of sequences with another identical sequence in the dataset per additional sequence (cyan). Points indicate the results from individual simulations and lines the LOESS curves.

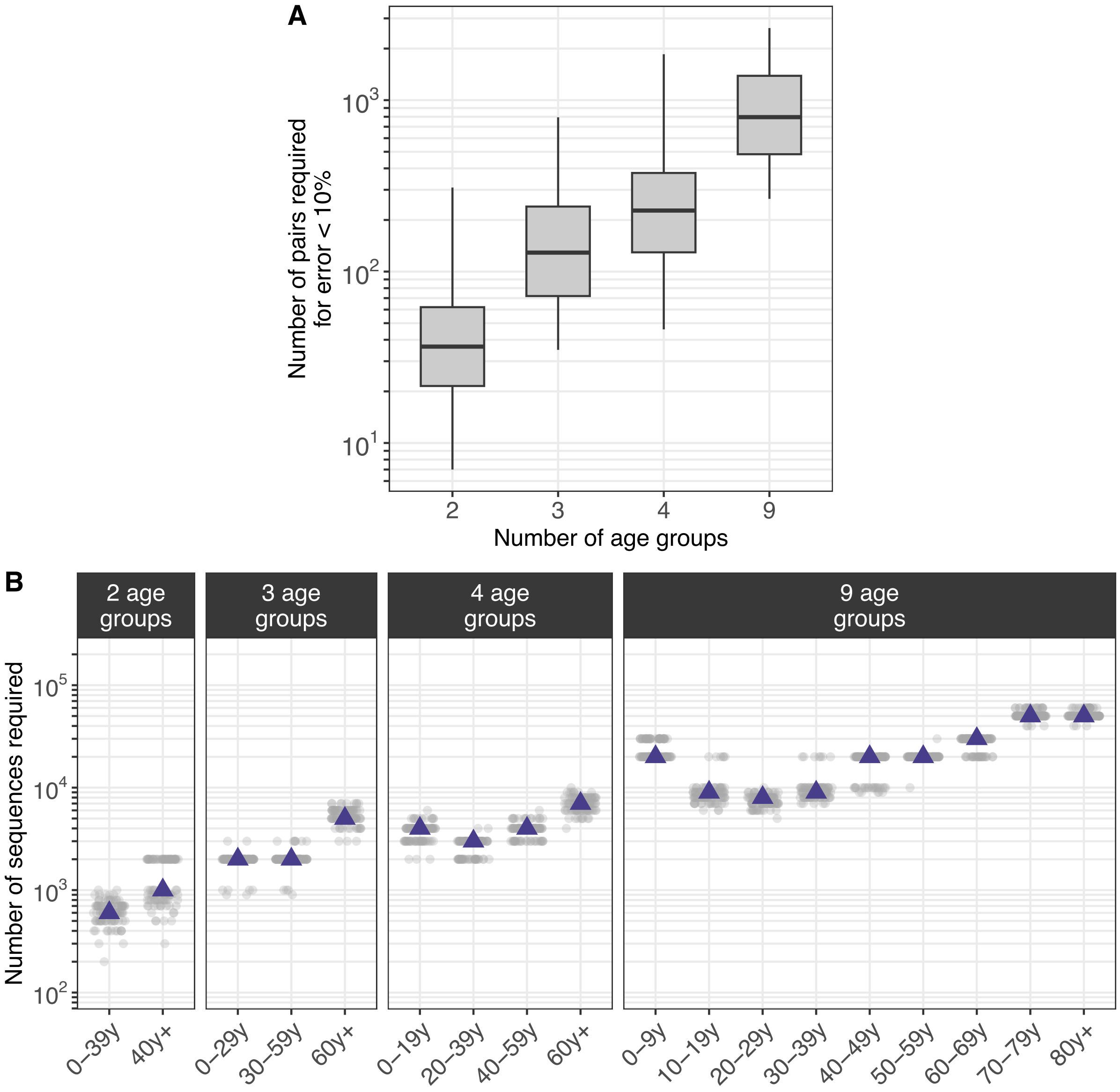

Figure S19. Impact of the number of groups included in the analysis on the dataset size required for the error in the relative risk of observing identical sequences to be lower than 10%. **A.** Number of pairs of identical sequences required for the error in the relative risk of observing identical sequences to be lower than 10%. Boxplots indicate the 2.5%, 25%, 50%, 75% and 97.5% percentiles. See Methods for a description of the downsampling strategy. **B.** Number of sequences required for the number of pairs of identical sequences observed within the age group on the x-axis to reach the median depicted in A. Each point corresponds to a subsampled dataset. Purple triangles indicate the median.

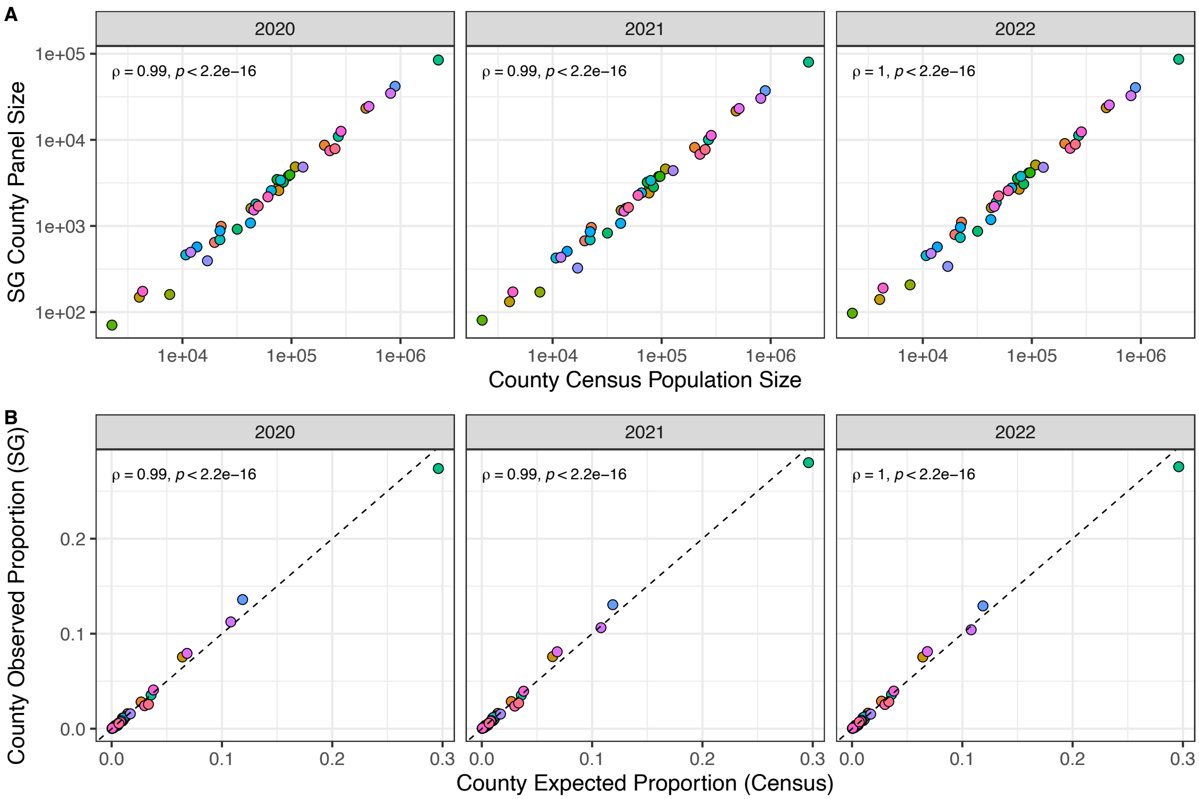

Figure S20. Comparisons between county census population sizes and SafeGraph panel sizes in Washington state, 2020 – 2022. A. County census population sizes strongly correlate with the mean number of devices tracked by SafeGraph ("SG") in each year. **B.** Expected proportions of devices based on county and state census population sizes strongly correlate with the observed proportion of devices tracked by SafeGraph ("SG") in each year. Points represent individual counties in WA state. In B, the black dashed line indicates the expected relationship for a true random sample of devices.

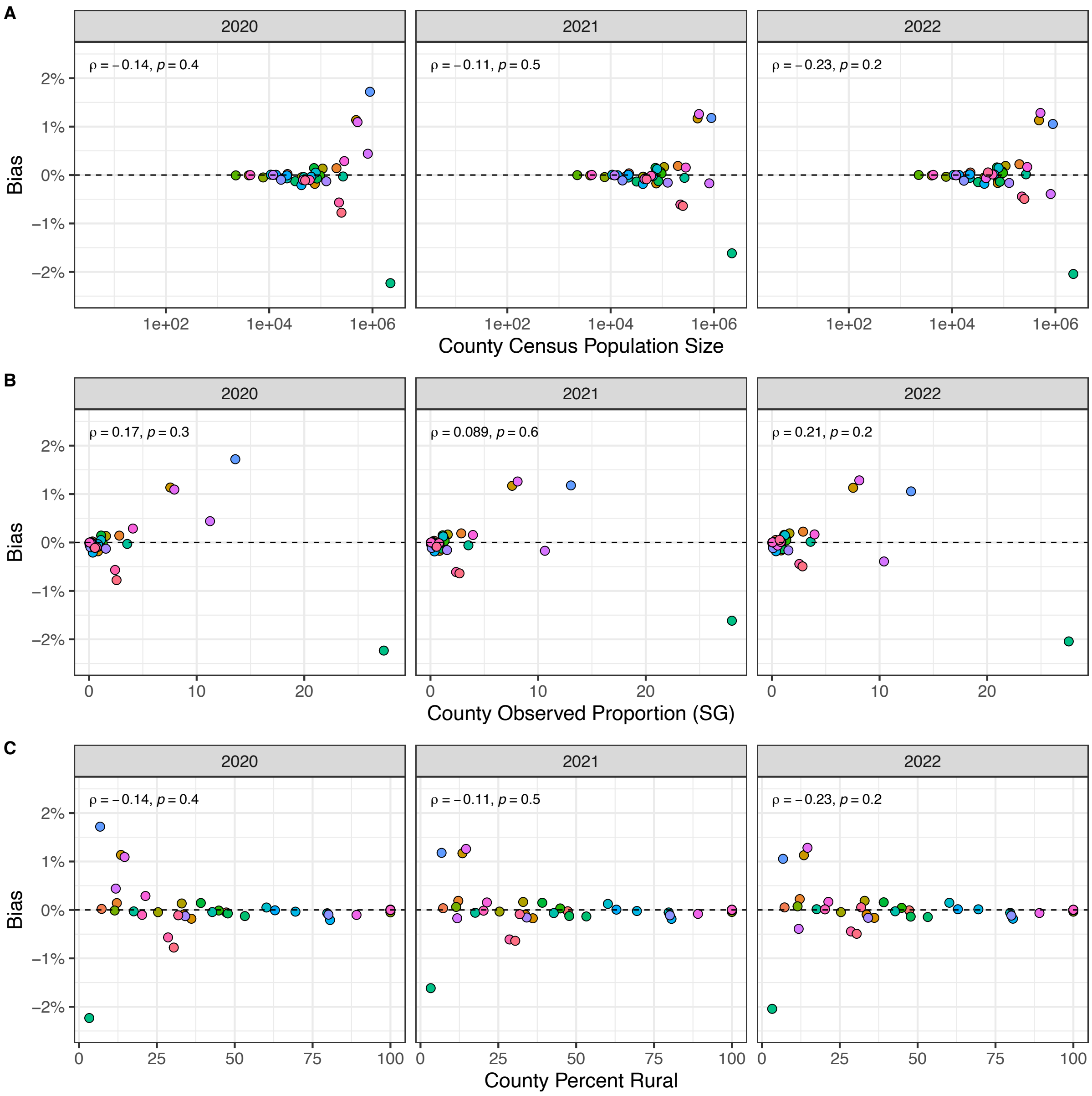

Figure S21. County-level bias of SafeGraph data in Washington state does not correlate with A. census population size, B. SafeGraph panel sizes in individual counties relative to WA state ("county observed proportion"), or C. census urban-rural classification, 2020 – 2022. Points represent individual counties in WA state. Bias is estimated as the "observed proportion" of devices tracked by SafeGraph in individual counties relative to WA state minus the "expected proportion" of devices based on census population sizes. Negative values indicate under-represented counties, and positive values indicate over-represented counties. The black dashed line (y = 0) indicates no bias in the SafeGraph panel of devices.

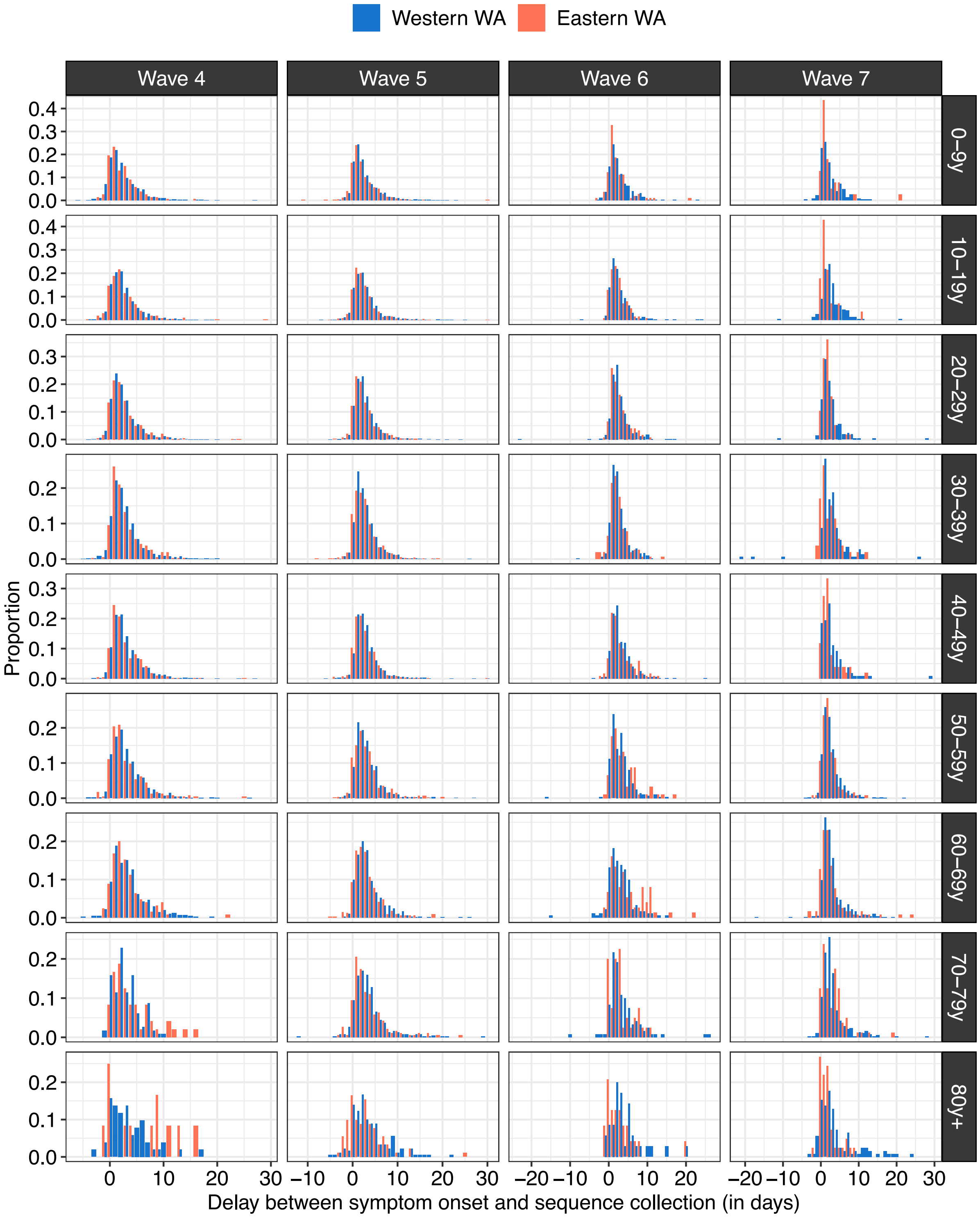

Figure S22. Empirical distribution of the delay between symptom onset and sequence collection by age (rows), period (columns) and geographic region (colours).

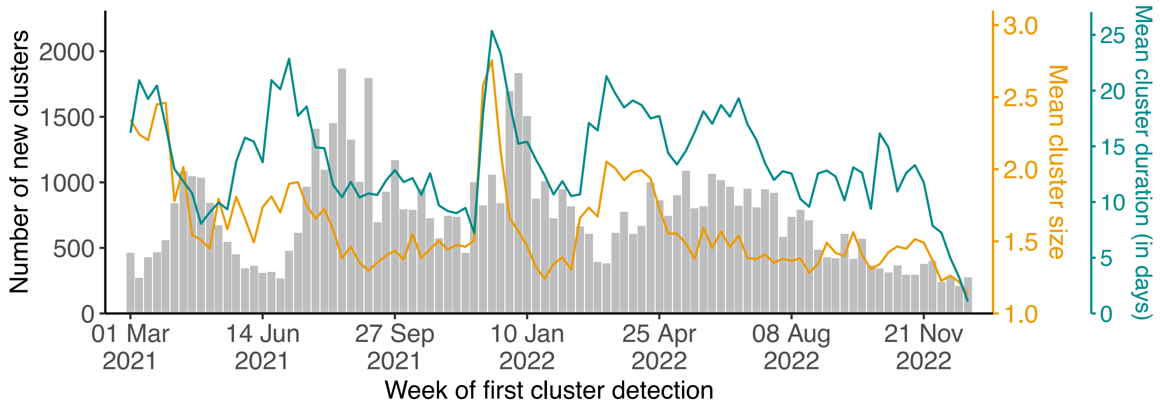

Figure S23. Characteristics of clusters of identical sequences across the study period. Grey bars (left y-axis) indicate the number of clusters by week of first cluster detection (defined as the week where the sequence with the earliest collection date was collected). The orange line (right orange y-axis) depicts the mean size of clusters of identical sequences by week of first cluster detection. The cyan line (right cyan y-axis) depicts the mean cluster duration by week of first cluster detection.

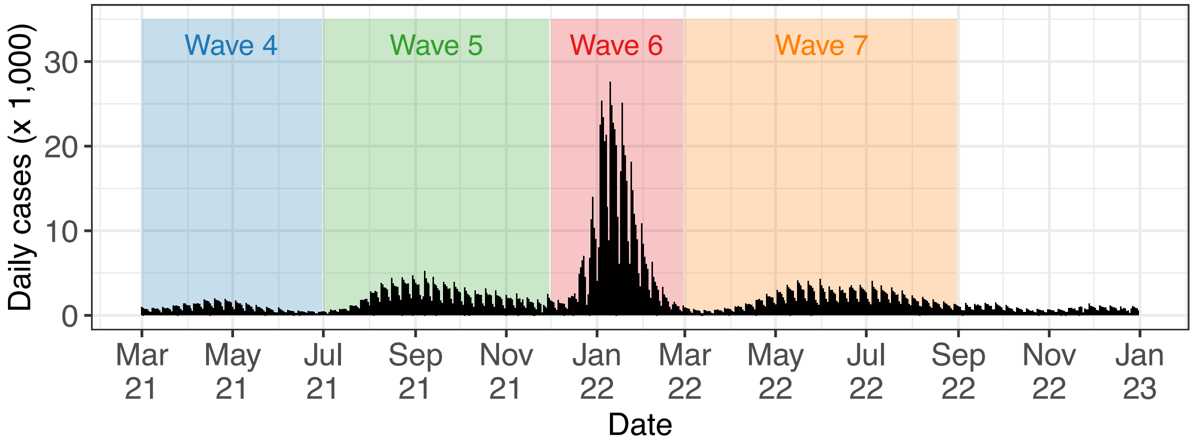

Figure S24. Time-series of COVID-19 cases in WA over the study periods. Shaded rectangles indicate the periods used to define the successive epidemic waves.

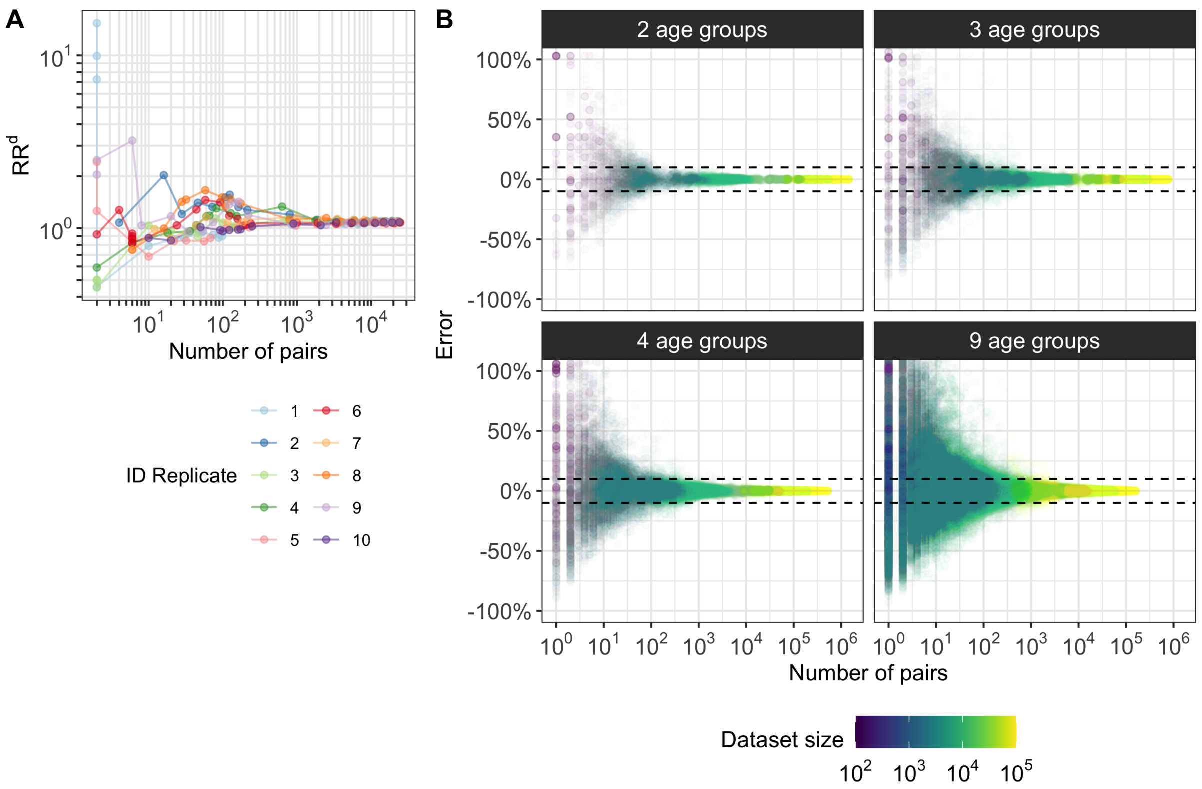

Figure S25. Illustration of the downsampling strategy used to quantify the amount of data required to compute relative risks. **A.** Relative risk RR^d of identical sequences being shared between the 0-9y computed on 10 downsampled datasets as a function of the number of pairs of identical sequences shared between the 0-9y. **B.** Error ϵ on the relative risk of observing identical sequences in downsampled datasets as a function of the number of pairs of identical sequences present for a pair of age groups in the downsampled datasets.

Method	Average sequencing probability	Replicate	Sample size (unbiased)	Sample size (biased)	Corr. with sim (unbiased)	Corr. with sim (biased)	Corr. biased / unbiased
**DTA** Fix tree	0.43 %	1	1745	1744	0.54	-0.22	-0.26
**DTA** Fix tree	0.43 %	2	1714	1805	0.60	0.19	0.35
**DTA** Fix tree	2.16 %	1	8723	8709	0.61	0.39	0.87
**DTA** Fix tree	2.16 %	2	8551	8675	0.77	0.25	0.70
**DTA** Inferring tree	0.43 %	1	1745	1744	0.10	0.15	-0.13
**RR**	8.66 %	1	34338	35304	0.91	0.74	0.77
**RR**	8.66 %	2	34736	35123	0.93	0.84	0.79
**RR**	2.16 %	1	8723	8709	0.94	0.80	0.79
**RR**	2.16 %	2	8551	8675	0.80	0.75	0.86

Table S1. Performance of Discrete Trait Analysis (DTA) and our relative risk metric (RR) in quantifying migration patterns. The sample sizes correspond to the number of sequences on which the inference is performed. All correlation coefficients reported are Spearman rank correlation coefficients. In the DTA analysis, we report the correlation between estimated and true migration rates (both for the biased and unbiased sequencing scenarios) and the correlation between the migration rates estimated on the biased and unbiased datasets. In the RR analysis, we report the correlation between the RR and the migration probability between demes (both for the biased and unbiased sequencing scenarios) as well as the correlation between the RR estimated on the biased and unbiased datasets.

Region	Adjacency status compared	p-value (Wilcoxon test)	p-value (Wilcoxon test) without 0
East-East	Within ZCTA & Adjacent ZCTAs	$5 \cdot 10^{-10}$	$3 \cdot 10^{-12}$
	Adjacent ZCTAs & Non-adjacent ZCTAs	$< 10^{-16}$	$< 10^{-16}$
East-West	Adjacent ZCTAs & Non-adjacent ZCTAs	0.73	0.39
West-West	Within ZCTA & Adjacent ZCTAs	$< 10^{-16}$	$< 10^{-16}$
	Adjacent ZCTAs & Non-adjacent ZCTAs	$< 10^{-16}$	$< 10^{-16}$

Table S2. Comparison of the relative of risk of observing identical sequences at the ZCTA level by adjacency level. We report the p-values of Wilcoxon rank sum test using either all pairs of ZCTAs or only pairs of ZCTAs for which pairs of identical sequences are collected (column "without 0").

Facility name	County	Prison capacity	County population size	Ratio prison capacity / county population
Washington State Penitentiary	Walla Walla	2439	62584	$3.90 \cdot 10^{-2}$
Stafford Creek Corrections Center	Grays Harbor	1936	75636	$2.56 \cdot 10^{-2}$
Coyote Ridge Corrections Center	Franklin	2468	96749	$2.55 \cdot 10^{-2}$
Washington Corrections Center	Mason	1268	65726	$1.93 \cdot 10^{-2}$
Clallam Bay Corrections Center	Clallam	858	77155	$1.11 \cdot 10^{-2}$
Airway Heights Corrections Center	Spokane	2258	539339	$4.19 \cdot 10^{-3}$
Olympic Corrections Center	Clallam	272	77155	$3.53 \cdot 10^{-3}$
Monroe Correctional Complex	Snohomish	2400	827957	$2.90 \cdot 10^{-3}$
Cedar Creek Corrections Center	Thurston	480	294793	$1.63 \cdot 10^{-3}$
Larch Corrections Center	Clark	240	503311	$4.77 \cdot 10^{-4}$

Table S3. Characteristics of WA male prisons.

Supplementary text S1: Relationship between the number of transmission pairs and the number of pairs with timing consistent with a transmission direction

Notations

Let $N_{X \to Y}$ denote the number of transmission pairs where the infector is in subgroup X and the infectee in subgroup Y. Let $N_{X<Y}$ denote the number of transmission pairs between subgroups X and Y (regardless of the transmission direction) where the timing of symptom onset is earlier in X than in Y. Let N_{XY} denote the total number of transmission pairs between subgroups X and Y (regardless of the transmission direction). Let $p_{X \to Y} = N_{X \to Y}/N_{XY}$ denote the proportion of transmission pairs between X and Y that were in the direction $X \to Y$. Let $p_{X<Y} = N_{X<Y}/N_{XY}$ denote the proportion of transmission pairs between X and Y where the timing of symptom onset is earlier in X than in Y.

We introduce p_0 as the proportion of transmission events with positive serial intervals (defined by the delay between the onset of symptom of the infectee and the infector).

Relationship between $p_{X<Y}$ and $p_{X \to Y}$

Here, we demonstrate that comparing $N_{X \to Y}$ and $N_{Y \to X}$ is equivalent to comparing $N_{X<Y}$ and $N_{Y<X}$ as long as p_0 is greater than 50%.

$$N_{X<Y} = N_{X \to Y} \cdot p_0 + N_{Y \to X} \cdot (1 - p_0)$$

By diving the two sides of this equation by N_{XY}, we have:

$$p_{X<Y} = p_{X \to Y} \cdot (2p_0 - 1) + (1 - p_0)$$

Therefore, if $p_0 > 0.5$,

$$\mathbf{p_{X<Y} > 0.5} \Leftrightarrow p_{X \to Y} \cdot (2p_0 - 1) + (1 - p_0) > 0.5 \Leftrightarrow p_{X \to Y} > \frac{p_0 - 0.5}{2p_0 - 1} \Leftrightarrow \mathbf{p_{X \to Y} > 0.5}$$

This means that as long as p_0 is greater than 0.5, comparing the number of transmission pairs between X and Y with symptom onset dates first occurring in X to the transmission pairs between X and Y with symptom onset dates first occurring in Y provides direct insights into the proportion of transmission pairs between X and Y happening in the $X \to Y$ direction.

Estimation of p_0 for SARS-CoV-2

For a given pathogen, p_0 can directly be estimated from a known serial interval distribution or from transmission pair data. For SARS-CoV-2, Geismar et al. [4] estimated this from reconstructed SARS-CoV-2 transmission events across a range of variants of concerns. In all analyses, their results show values greater of p_0 greater than 50 %.

Here, we focus on the timing of symptom onset within transmission pairs. However, this argument is directly transposable to other timing definitions, such as the timing of sequence collection dates by replacing p_0 by the proportion of transmission pairs where the sequence collection date of the infectee occurs after the sequence collection date of the infector.

References

[1] US Census Bureau. 2010 Census County, 2010 TIGER/Line Shapefile. `https://ofm.wa.gov/washington-data-research/population-demographics/gis-data/census-geographic-files`

[2] United States Census Bureau. Commuting flows. `https://www.census.gov/topics/employment/commuting/guidance/flows.html`.

[3] Cécile Tran-Kiem and Trevor Bedford. Estimating the reproduction number and transmission heterogeneity from the size distribution of clusters of identical pathogen sequences. *Proc. Natl. Acad. Sci. U. S. A.*, 12(15):e2305299121, 2024.

[4] Cyril Geismar, Vincent Nguyen, Ellen Fragaszy, Madhumita Shrotri, Annalan M D Navaratnam, Sarah Beale, Thomas E Byrne, Wing Lam Erica Fong, Alexei Yavlinsky, Jana Kovar, Susan Hoskins, Isobel Braithwaite, Robert W Aldridge, Andrew C Hayward, Peter J White, Thibaut Jombart, and Anne Cori. Bayesian reconstruction of SARS-CoV-2 transmissions highlights substantial proportion of negative serial intervals. *Epidemics*, 44:100713, 2023.
