# Supplementary material for: Fine-scale patterns of SARS-CoV-2 spread from identical pathogen sequences
Source: Nature. 2025 Mar 5;640(8057):176–85. doi: 10.1038/s41586-025-08637-4 (PMC11964829; doi:10.1038/s41586-025-08637-4)
Supplement: Supplementary file 2 — Reporting Summary [file 41586_2025_8637_MOESM2_ESM.pdf]

nature portfolio

Corresponding author(s): Cecile Tran Kiem

Last updated by author(s): Dec 11, 2024

Reporting Summary

Nature Portfolio wishes to improve the reproducibility of the work that we publish. This form provides structure for consistency and transparency in reporting. For further information on Nature Portfolio policies, see our Editorial Policies and the Editorial Policy Checklist.

Statistics

For all statistical analyses, confirm that the following items are present in the figure legend, table legend, main text, or Methods section.

n/a	Confirmed	
☒	☐	The exact sample size (n) for each experimental group/condition, given as a discrete number and unit of measurement
☐	☒	A statement on whether measurements were taken from distinct samples or whether the same sample was measured repeatedly
☐	☒	The statistical test(s) used AND whether they are one- or two-sided<br>*Only common tests should be described solely by name; describe more complex techniques in the Methods section.*
☐	☒	A description of all covariates tested
☒	☐	A description of any assumptions or corrections, such as tests of normality and adjustment for multiple comparisons
☐	☒	A full description of the statistical parameters including central tendency (e.g. means) or other basic estimates (e.g. regression coefficient) AND variation (e.g. standard deviation) or associated estimates of uncertainty (e.g. confidence intervals)
☐	☒	For null hypothesis testing, the test statistic (e.g. F, t, r) with confidence intervals, effect sizes, degrees of freedom and P value noted<br>*Give P values as exact values whenever suitable.*
☒	☐	For Bayesian analysis, information on the choice of priors and Markov chain Monte Carlo settings
☒	☐	For hierarchical and complex designs, identification of the appropriate level for tests and full reporting of outcomes
☐	☒	Estimates of effect sizes (e.g. Cohen's d, Pearson's r), indicating how they were calculated

Our web collection on statistics for biologists contains articles on many of the points above.

Software and code

Policy information about availability of computer code

Data collection	Sequence data were downloaded from the GISAID EpiCov using the Nextstrain ncov-ingest pipeline. These sequences are publicly available with standard metadata (generally consisting of date of sample collection and sometimes county of sample collection). More detailed metadata curated by Washington State Department of Health (WA DOH) of county, postal code, age group and vaccination status were shared with the Fred Hutchinson Cancer Center.
Data analysis	Data were analysed using custom R scripts available at: https://github.com/blab/phylo-kernel-public/. The scripts were also deposited on Zenodo at the following DOI: https://doi.org/10.5281/zenodo.14398514

For manuscripts utilizing custom algorithms or software that are central to the research but not yet described in published literature, software must be made available to editors and reviewers. We strongly encourage code deposition in a community repository (e.g. GitHub). See the Nature Portfolio guidelines for submitting code & software for further information.

Data

Policy information about availability of data

All manuscripts must include a data availability statement. This statement should provide the following information, where applicable:

- Accession codes, unique identifiers, or web links for publicly available datasets
- A description of any restrictions on data availability
- For clinical datasets or third party data, please ensure that the statement adheres to our policy

All sequences referenced in this manuscript are publicly shared to GISAID and are publicly available with standard metadata (generally consisting of date of sample collection and sometimes county of sample collection).
More detailed metadata curated by Washington State Department of Health (WA DOH) of county, postal code, age group and vaccination status were shared with the Fred Hutchinson Cancer Center under a Data Sharing Agreement for Confidential Data with an associated IRB.
These more detailed metadata are not currently shared publicly while we seek clearance with WA DOH.
GISAID accessions and a sequence-level acknowledgements table are provided in the GitHub repository associated with this manuscript. They were also deposited on Zenodo at the following DOI: https://doi.org/10.5281/zenodo.14398514.

Research involving human participants, their data, or biological material

Policy information about studies with human participants or human data. See also policy information about sex, gender (identity/presentation), and sexual orientation and race, ethnicity and racism.

Reporting on sex and gender	Data are not analysed by sex and gender.
Reporting on race, ethnicity, or other socially relevant groupings	Data are not analysed by race or ethnicity.
Population characteristics	We included all SARS-CoV-2 sequences collected through WA state genomic sentinel surveillance between 1 March 2021 and 31 December 2022. The age of individuals was grouped as: 0-9y, 10-19y, 20-29y, 30-29y, 40-49y, 50-59y, 60-69y, 70-79y and 80y+.
Recruitment	Sequence data were collected as part of Washington State genomic sentinel surveillance. The implementation of this surveillance system is described in: 10.3201/eid2902.221482.
Ethics oversight	The Washington State and University of Washington Institutional Review Boards determined this project to be surveillance activity and exempt from review; the need for informed consent was waived through this determination.<br>Under Washington State IRB Exempt Determination 2020-102, symptom onset date, age group, residence county, residence postal code and vaccination history was provided by the Washington Department of Health from the Washington Disease Reporting System for individuals with linked sequenced SARS-CoV-2 samples from March 1, 2021 through December 31, 2022. Sequencing and analysis of samples from the Seattle Flu Study was approved by the Institutional Review Board (IRB) at the University of Washington (protocol STUDY00006181). Sequencing of remnant clinical specimens at UW Virology Lab was approved by the University of Washington Institutional Review Board (protocol STUDY00000408).

Note that full information on the approval of the study protocol must also be provided in the manuscript.

Field-specific reporting

Please select the one below that is the best fit for your research. If you are not sure, read the appropriate sections before making your selection.

☒ Life sciences ☐ Behavioural & social sciences ☐ Ecological, evolutionary & environmental sciences

For a reference copy of the document with all sections, see nature.com/documents/nr-reporting-summary-flat.pdf

Life sciences study design

All studies must disclose on these points even when the disclosure is negative.

Sample size	We used all available sequences from WA sentinel surveillance between 1 March 2021 and 31 December 2022 with linked metadata (116,791 sequences).
Data exclusions	When multiple sequences were linked to a de-identified patient ID, we only included the earliest sequence collected (for each of the patient with multiple sequences). This was done to avoid including sequences from the same individual and comparing individual to themselves (as these sequences are very likely to be identical when sampled during the same time period). This resulted in 114,306 SARS-CoV-2 sequences with unique patient ID.
Replication	NA
Randomization	NA

Blinding	NA

Reporting for specific materials, systems and methods

We require information from authors about some types of materials, experimental systems and methods used in many studies. Here, indicate whether each material, system or method listed is relevant to your study. If you are not sure if a list item applies to your research, read the appropriate section before selecting a response.

Materials & experimental systems

n/a	Involved in the study
☒	☐ Antibodies
☒	☐ Eukaryotic cell lines
☒	☐ Palaeontology and archaeology
☒	☐ Animals and other organisms
☒	☐ Clinical data
☒	☐ Dual use research of concern
☒	☐ Plants

Methods

n/a	Involved in the study
☒	☐ ChIP-seq
☒	☐ Flow cytometry
☒	☐ MRI-based neuroimaging

Plants

Seed stocks	NA
Novel plant genotypes	NA
Authentication	NA
